# Meta-analytic Review of Memory Impairment in Behavioral Variant Frontotemporal Dementia

**DOI:** 10.1017/S1355617718000115

**Published:** 2018-03-19

**Authors:** Jackie M. Poos, Lize C. Jiskoot, Janne M. Papma, John C. van Swieten, Esther van den Berg

**Affiliations:** 1 Alzheimer Center and Department of Neurology, Erasmus University Medical Center, Rotterdam, the Netherlands; 2 Department of Radiology, Leiden University Medical Center, Leiden, the Netherlands; 3 Department of Clinical Genetics, VU Medical Center, Amsterdam, the Netherlands

**Keywords:** Episodic memory, Learning, Recall, Alzheimer’s disease, bvFTD; Meta-analysis

## Abstract

**Objectives:** A meta-analysis of the extent, nature and pattern of memory performance in behavioral variant frontotemporal dementia (bvFTD). Multiple observational studies have challenged the relative sparing of memory in bvFTD as stated in the current diagnostic criteria. **Methods:** We performed a meta-analytic review covering the period 1967 to February 2017 of case-control studies on episodic memory in bvFTD *versus* control participants (16 studies, 383 patients, 603 control participants), and patients with bvFTD *versus* those with Alzheimer’s disease (AD) (20 studies, 452 bvFTD, 874 AD). Differences between both verbal and non-verbal working memory, episodic memory learning and recall, and recognition memory were examined. Data were extracted from the papers and combined into a common metric measure of effect, Hedges’ *d*. **Results:** Patients with bvFTD show large deficits in memory performance compared to controls (Hedges’ *d* –1.10; 95% confidence interval [CI] [–1.23, –0.95]), but perform significantly better than patients with AD (Hedges’ *d* 0.85; 95% CI [0.69, 1.03]). Learning and recall tests differentiate best between patients with bvFTD and AD (*p*<.01). There is 37–62% overlap in test scores between the two groups. **Conclusions:** This study points to memory disorders in patients with bvFTD, with performance at an intermediate level between controls and patients with AD. This indicates that, instead of being an exclusion criterion for bvFTD diagnosis, memory deficits should be regarded as a potential integral part of the clinical spectrum. (*JINS*, 2018, *24*, 593–605)

## INTRODUCTION

Frontotemporal dementia (FTD) is an early-onset dementia characterized by a heterogeneous clinical presentation including behavioral changes, frontal-executive deficits, and/or language disorders (Seelaar, Rohrer, Pijnenburg, Fox, & van Swieten, 2011), caused by pathophysiological damage in the frontal and temporal lobes (McKhann et al., 2001; Rohrer & Rosen, 2013). Behavioral variant FTD (bvFTD) is the most common clinical syndrome in the spectrum and is associated with deficits in social cognition and executive functioning. Patients with bvFTD frequently exhibit impaired theory of mind, emotional processing, fluency, planning, set shifting, and working memory (e.g., Bora, Walterfang, & Velakoulis, 2015; Hornberger, Piquet, Kipps, & Hodges, 2008; van den Berg, Jiskoot, Grosveld, van Swieten, & Papma, 2017). Day-to-day memory is thought to be relatively preserved in the early stage of the disease (Rascovsky et al., 2011; Rosen et al., 2002), with severe memory impairment as exclusion criterion. However, many patients with bvFTD have self-reported or caregiver reported memory problems (Hornberger & Piguet, 2012) and some patients even manifest severe episodic memory disorders, even at initial presentation (e.g., Hornberger, Piguet, Graham, Nestor, & Hodges, 2010; Shi et al., 2005).

Systematic investigations of episodic memory functioning in patients with bvFTD are scarce (Hornberger & Piguet, 2012) and inconsistent, with some studies revealing no differences between bvFTD and Alzheimer’s disease (AD) memory performance (e.g., Gregory, Orrell, Sahakian, & Hodges, 1997; Hodges et al., 1999; Walker, Meares, Sachdev, & Brodaty, 2005), and others demonstrating a relative sparing of memory performance in bvFTD compared to AD (e.g., Frisoni et al., 1995; Pachana, Boone, Miller, Cummings, & Berman, 1996; Thompson, Stopford, Snowden, & Neary, 2005). Studies showing memory impairment in patients with bvFTD suggest poor organization and a lack of efficient learning and retrieval strategies as causes (i.e., dysexecutive syndrome), rather than deficits in memory consolidation *per se* (Blumenfeld & Ranganath, 2007; Pasquier, Grymponprez, Lebert, & Van der Linden, 2001; Wang & Miller, 2007).

In line with the latter, there are indications that patients with bvFTD and AD will not differ on delayed memory testing, but that they will benefit more from cued or recognition memory formats (e.g., Glosser, Gallo, Clark, & Grosmann, 2002). However, specific differential memory processes have, as of yet, not been studied consistently in bvFTD. Involvement of the hippocampal structures, as found in neuroimaging studies of both FTD and AD, suggests that amnesia in bvFTD may be due to real defects in memory storage and consolidation processing (e.g., Hornberger et al., 2012; Lindberg et al., 2012; Munoz-Ruiz et al., 2012; Papma et al., 2013; de Souza et al., 2013). For example, Papma and colleagues (2013) showed lower perfusion in the right temporal lobe in amnesic patients with FTD compared to non-amnesic patients with FTD (Papma et al., 2013). The authors argue that amnesic patients with FTD might represent an anatomical subtype of FTD, with prominent right temporal lobe involvement.

A possible explanation for these contrasting results is the lack of pathological confirmation in most studies. Some have included patients with possible or probable FTD, whereas only a few have looked at memory disorder in pathological confirmed FTD (e.g., *post-mortem*, genotyping, or excluding AD biomarkers)(Rascovsky et al., 2011). Those studies that have looked at memory disorder in pathological confirmed FTD show clear episodic memory deficits (e.g., Bertoux et al., 2014; Graham et al., 2005; Hornberger et al., 2011). For the differential diagnosis between bvFTD and AD, it is important that the presence of memory impairment is not exclusively related to AD, but that it may also be included in the diagnosis of bvFTD. Clarifying the patterns of specific memory processes in both groups could help differentiate AD and bvFTD.

The primary aim of the present meta-analysis was to quantify the nature and extent of memory impairment in patients with bvFTD compared to AD and control participants. We examined the proposed contrasts in differential memory processes (working memory, episodic memory learning and recall, and recognition memory) to provide further insights into the pattern of memory impairment in bvFTD. In addition, we tested the occurrence of differences in memory disorders between the studies, including possible, probable or definite diagnoses. By quantifying the nature and extent of bvFTD memory impairment, we provide insights into how memory performance in clinical evaluation can help in differential diagnostics between patients with bvFTD and AD.

## METHODS

### Identification of Studies

The meta-analysis included all published studies that provide an estimate of memory performance in patients with bvFTD. Studies were selected by means of a Medline literature search covering the period April 1967 to February 18, 2017. Key search terms were (“frontotemporal dementia” or “frontal dementia” or “Pick’s disease” or “frontotemporal lobe dementia” or “frontal lobe dementia” or “dementia of the frontal type”) in combination with (“memory” or “learning” or “cognition” or “neuropsychology” and its derivatives) in full or truncated versions. Titles and abstracts were scanned and potentially eligible papers were collected in full-text.

In addition, lists of references of these studies were examined for additional papers. To be selected for the meta-analysis, a study had to meet the following inclusion criteria: (1) the study was an original English language article; (2) memory performance was assessed in both a bvFTD patient group and healthy control participants or an AD patient group, all with a group size of *n* ≥ 10 and matched for demographic variables age and level of education; (3) raw test scores were presented for the patient and the control participant groups (i.e., means and standard deviations).

To prevent including the same cohorts of patients across studies, of all the eligible studies (bvFTD *vs*. healthy controls 26 studies; and bvFTD *vs*. AD 24 studies), we included the study that had the largest sample and/or included the most detailed memory assessment per cohort for each center. If studies did not specify from which cohort patients were included, only one study per center was selected. Sixteen validated memory measurements were included (see Tables 1 and 2) with tasks typically involving the presentation of either verbal or visual information in which participants have several trials to memorize the presented items, including immediate and delayed recall trials. Our study was conducted in accordance with the Helsinki Declaration and followed the PRISMA guidelines for systematic reviews and meta-analyses (Moher, Liberati, Tetzlaff, & Altman, 2009). Since we only reviewed previously published data, no additional medical ethical approval was necessary.

**Table 1:** Study characteristics of studies included in the meta-analysis: bvFTD versus control participants

	*n*		Age		Gender (% female)		Education (yrs)		MMSE			
Study	F	C	F	C	F	C	F	C	F	C	Dementia diagnosis	Memory measurements
Mandelli et al. (2016)	23	34	62.9 (6.5)	62.3 (6.6)	43	65	16.1 (2.6)	16.4 (2.1)	26.6 (3.5)	28.4 (1.2)	Rascovsky et al. (2011)	CVLT-SF, RCFT, digit span
Balconi et al. (2015)	16	20	65.6 (6.9)	68.6 (4.5)	13	50	7 (2.2)	7.8 (2.7)	25.3 (3.3)	28.6 (1)	Neary et al. (1998)	Logical memory, RCFT
Hardy et al. (2015)	24	24	64.6 (7.7)	63.8 (7.8)	17	63	14.8 (3.8)	15.3 (2.9)	24 (5.7)	30 (0.6)	Rascovsky et al. (2011)	RMT F/W, digit span
Tu et al. (2015)	24	23	64.7 (9.3)	68 (3.4)	26	52	11.8 (3.1)	13.3 (3.1)	N.S	N.S.	Rascovsky et al. (2011)	RAVLT, RCFT, digit span
Smits et al. (2015)	20	112	63 (8)	61 (8)	49	56	5.0 (1.3)^a^	5.5 (1.1)^a^	26 (3)	28 (1)	Rascovsky et al. (2011)	RAVLT, VAT
Lemos et al. (2014)	32	32	68.6 (1.2)	68.6 (1.3)	31	31	6.9 (0.8)	7 (0.9)	26.9 (0.4)	29.1 (0.2)	Rascovsky et al. (2011)	FCSRT, digit span, BVMT-R
Bertoux et al. (2014)	44	22	66.9 (8.3)	66.7 (9.3)	43	41	10.8 (3.9)	12.8 (2.4)	23.1 (3.6)	29 (2.6)	Rascovsky et al. (2011)	FCSRT
Virani et al. (2013)	14	17	65.3 (8.1)	62.4 (10.8)	25	28	11.3 (3.0)	15.1 (3.5)	20.6 (6.9)	27.1 (4.2)	Rascovsky et al. (2011)	prose memory
Ricci et al. (2012) - Italian	15	28	65.7 (8.6)	68.5 (6.7)	40	61	10.3 (3.2)	11.4 (2.8)	26.6 (3.4)	N.S.	Neary et al. (1998)	RAVLT
Ricci et al. (2012) - Australian	11	15	59.8 (9)	60.2 (5.8)	55	60	12.6 (2.4)	13.5 (3.0)	26.5 (2.3)	N.S.	Neary et al. (1998)	RAVLT
Stopford et al. (2012)	26	26	64 (6)	59 (13.5)	50	69	N.S.	N.S.	23 (6)	N.S.	Neary et al. (1998)	Logical memory, digit span
Giovagnoli et al. (2008)	40	91	61.1 (10.7)	62.3 (10)	38	55	8.9 (4.1)	11.3 (4.4)	N.S	N.S.	Neary et al. (1998)	The short story, RCFT, digit span, Corsi cube span
Piolino et al. (2007)	13	21	67.2 (7.9)	69.9 (8.6)	N.S.	N.S.	N.S.	N.S.	24.8 (4)	N.S.	Neary et al. (1998)	Grober & Buscke Test, digit span
Torralva et al. (2007)	20	10	67.2 (8.1)	63.5 (5.8)	45	60	12.8 (5)	13.5 (2.7)	27.9 (1.6)	29.5 (0.8)	Neary et al. (1998)	Logical memory, digit span
Wicklund et al. (2006)	20	48	61.9 (8.4)	72.1 (7.2)	30	79	14.7 (2.9)	15.2 (2.7)	23.8 (4.7)	29.2 (0.9)	Neary et al. (1998)	WMS-R, CERAD
Clague et al. (2005)	11	41	60.3 (6.9)	64.9 (6.1)	N.S.	N.S.	12.4 (1.8)	12.8 (2.2)	27.1 (2.0)	N.S.	Neary et al. (1998)	Digit span, logical memory, RCFT
Gregory et al. (2002)	19	16	58.6 (6.9)	57.1 (5.1)	16	50	11.6 (2.2)	12.1 (1.5)	26.6 (3.2)	28.7 (1)	Neary et al. (1998)	Digit span, logical memory, RCFT

F=bvFTD; C=control participants; MMSE=Mini Mental State Examination; CVLT-SF=California Verbal Learning Test – Short version; RCFT=Rey Complex Figure Test; RMT F/W=Recognition Memory Test Words/Faces; RAVLT=Rey Auditory Verbal Learning Test; VAT=Visual Association Test; FCSRT=Free and Cued Selective Reminding Test; BVMT-R=Brief Visuospatial Memory Test- Revised; WMS-R=Wechsler Memory Scale – Revised; CERAD=Consortium to Establish a Registry for Alzheimer’s Disease; N.S.=not specified.

a According to the Verhage system.

**Table 2:** Study characteristics of studies included in the meta-analysis: bvFTD versus AD

	*n*		Age		Gender (% female)		Education (yrs)		MMSE		Dementia diagnosis		
Study	F	A	F	A	F	A	F	A	F	A	F	A	Memory measurements
Balconi et al. (2015)	16	14	65.6 (6.9)	72.2 (6.9)	13	71	7 (2.2)	7.2 (3.8)	25.3 (3.3)	21.1 (4.2)	Neary et al. (1998)	NINCDS-ADRDA	Logical memory, RCFT
Smits et al. (2015)	20	199	63 (8)	65 (8)	49	44	5.0 (1.3)^a^	4.9 (1.2)^a^	26 (3)	22 (4)	Rascovsky et al. (2011)	NINCDS-ADRDA	RAVLT, VAT
Tu et al. (2015)	24	23	64.7 (9.3)	68 (3.4)	26	52	11.8 (3.1)	13.3 (3.1)	N.S.	N.S.	Rascovsky et al. (2011)	NINCDS-ADRDA	RAVLT, RCFT, digit span
Barsuglia et al. (2014)	16	18	61.1 (10.6)	59.2 (4.9)	50	67	15.6 (2.3)	16.2 (2.3)	24.6 (4.3)	24.4 (4.6)	Rascovsky et al. (2011)	NINCDS-ADRDA	Digit span, logical memory RCFT
Lemos et al. (2014)	32	32	68.6 (1.2)	69.7 (1.3)	31	47	6.9 (0.8)	6.9 (0.9)	26.9 (0.4)	21.2 (0.7)	Rascovsky et al. (2011)	NINCDS-ADRDA	FCSRT, digit span, BVMT-R
Perri et al. (2014)	21	22	64.7 (11.5)	66.8 (3.5)	55	48	11.2 (3.4)	10.5 (5.4)	23.4 (2.8)	23.2 (1.9)	Neary et al. (1998)	NINCDS-ADRDA	15 word list recall, prose memory
Bertoux et al. (2014)	44	56	66.9 (8.3)	66.4 (9.4)	43	57	10.8 (3.9)	12.3 (3.5)	23.1 (3.6)	21.9 (4.4)	Rascovsky et al. (2011)	N.S.	FCSRT
Ricci et al. (2012) - Italian	15	39	65.7 (8.6)	68.3 (7.7)	40	54	10.3 (3.2)	12.1 (3.2)	26.6 (3.4)	23.5 (3.7)	Neary et al. (1998)	NINCDS-ADRDA	RAVLT
Ricci et al. (2012) - Australian	11	17	59.8 (9)	65.3 (7.8)	55	59	12.6 (2.4)	12.3 (3.5)	26.5 (2.3)	24.4 (3.5)	Neary et al. (1998)	NINCDS-ADRDA	RAVLT
Mendez & Shapira (2011)	12	12	63.4 (4.4)	64.6 (4.8)	50	50	14.5 (4.1)	14.2 (4.3)	24.3 (2.8)	22.6 (4.1)	Rascovsky et al. (2011)	N.S.	Digit span, CERAD
Giovagnoli et al. (2008)	40	77	61.1 (10.7)	65.5 (9.9)	38	63	8.9 (4.1)	8.9 (4.9)	N.S.	N.S.	Neary et al. (1998)	NINCDS-ADRDA	The short story, RCFT, digit span, Corsi cube span
Heidler-Gary et al. (2007)	25	30	64.8 (9.9)	72.6 (8.8)	N.S.	N.S.	N.S.	N.S.	N.S.	18.9 (5.4)	Neary et al. (1998)	NINCDS-ADRDA	RAVLT
Luzzi et al. (2007)	11	14	64 (7)	71 (8)	27	50	10 (2)	10(4)	24 (6)	24 (2)	Neary et al. (1998)	NINCDS-ADRDA	Digit span, Hopkins test, RAVLT, RCFT
Castiglioni et al. (2006)	33	85	69.8 (8.8)	74.4 (7.4)	33	64	8 (4.8)	7.3 (4.0)	20.8 (3.8)	19.4 (3)	Neary et al. (1998)	NINCDS-ADRDA	Story memory, RCFT, Corsi span, digit span
Wicklund et al. (2006)	20	33	61.9 (8.4)	72.6 (9.6)	40	67	14.7 (2.9)	14.2 (2.8)	23.8 (4.7)	23.6 (4)	Neary et al. (1998)	NINCDS-ADRDA	WMS-R, CERAD
Clague et al. (2005)	11	14	60.3 (6.9)	63.8 (6.9)	N.S.	N.S.	12.4 (1.8)	11 (1.2)	27.1 (2.0)	24.5 (2)	Neary et al. (1998)	NINCDS-ADRDA	Digit span, logical memory, RCFT
Glosser et al. (2002)	12	30	68.8 (10.1)	73.7 (6.8)	67	47	15.1 (3.1)	14.8 (3.2)	23.8 (2.3)	23.6 (4.6)	Neary et al. (1998)	NINCDS-ADRDA	CVLT, BFLT
Gregory et al. (2002)	19	12	58.6 (6.9)	66.5 (8.9)	16	50	11.6 (2.2)	14.4 (4)	26.6 (3.2)	27.1 (1.7)	Neary et al. (1998)	NINCDS-ADRDA	Digit span, logical memory, RCFT
Siri et al. (2001)	14	14	74.8 (7.3)	69.6 (7.4)	N.S.	N.S.	5.3 (2.8)	7.2 (2.9)	17.8 (4.2)	20.3 (5.8)	Neary et al. (1998)	NINCDS-ADRDA	Digit span, Corsi block span Story memory, RCFT
Binetti et al. (2000)	44	121	66.9 (9.2)	70.6 (8.5)	41	62	14.1 (3.5)	13.1 (3.5)	N.S.	N.S.	DSM-IV, ICD-10	N.S.	Story memory, figure recall, BVRT
Gregory et al. (1997)	12	12	63.6	71.1	N.S.	N.S.	11.7	9.9	25.3	24.1	Neary et al. (1998)	NINCDS-ADRDA	Story memory, RCFT, Corsi block span, digit span

*Note.* Abbreviations as in Table 1.

F=bvFTD; A=AD; MMSE=Mini Mental State Examination; CVLT-SF=California Verbal Learning Test−Short version; RCFT=Rey Complex Figure Test; RMT F/W=Recognition Memory Test Words/Faces; RAVLT=Rey Auditory Verbal Learning Test; VAT=Visual Association Test; FCSRT=Free and Cued Selective Reminding Test; BVMT-R=Brief Visuospatial Memory Test- Revised; WMS-R=Wechsler Memory Scale−Revised; CERAD=Consortium to Establish a Registry for Alzheimer’s Disease; BVRT=Benton Visual Retention Test; BFLT=Biber Figure Learning Test; N.S.=not specified.^a^According to the Verhage system.

### Data Synthesis and Analysis

Effect sizes were calculated for the difference in test scores between (1) patients with bvFTD and healthy control participants, and (2) patients with bvFTD and AD. We used Hedges’ *d* (the standardized difference between the groups) to estimate effect size (Hedges & Olkins, 1985). We chose Hedges’ *d* instead of Cohen’s *d* or Hedges’ *g* as it corrects for bias due to small sample sizes (Hedges & Olkins, 1985). The direction of the effect size was negative if the performance of the bvFTD patient group was worse than the control or AD patient group. In the meta-analysis, an overall *d* value was calculated, expressing the magnitude of associations across studies weighted for sample size (Hedges & Olkin, 1985).

According to Cohen’s nomenclature (1988), *d*>0.80 indicates a large difference. A bias-corrected 95% confidence interval (CI) was calculated based on the standard error. The percentage of overlap in test scores between groups was also reported according to Zakzanis’ calculations (2001); *d*=0 equates to 100% overlap, *d*=1.0 equates to 45% overlap and *d*=3.0 equates to less than 5% overlap in group scores. In addition, the overall effect size was used in a random effects model to determine the total heterogeneity of effect sizes (*Q*_*T*_) and tested against the χ^2^ distribution with n-1 degrees of freedom (Hedges & Olkin, 1985). A significant *Q*_*T*_ means that the variance of the effect sizes is greater than expected from sampling errors and suggests that other explanatory variables should be investigated.

The differences between the overall effect sizes of the memory processes (working memory, episodic memory learning recall, and recognition memory) were examined with the Q-statistic for heterogeneity. This procedure is analogous to analysis of variance, where a difference among group means is determined. We partitioned the total heterogeneity *Q*_*T*_ in *Q*_*M*_, which is the variation in effect sizes explained by the model, and *Q*_*E*_, which is the residual error variance not explained by the model. *Q*_*M*_ is thus a description of the difference among group cumulative effect sizes, and a significant *Q*_*M*_ suggests a difference between the overall effect sizes for the different memory processes (Hedges & Olkin, 1985). The fail-safe number was computed to explore the robustness of the results to publication bias. The fail-safe number of studies *N*_*R*_ provides an estimation of how many non-significant or missing studies would be needed to render the observed meta-analytical results non-significant (Rosenthal’s method: α<0.05; Rosenthal & DiMatteo, 2001).

All analyses were performed in MetaWin 2.0 (Rosenberg, Adams, & Gurevitch, 2000). Data for the different memory processes were separately included in the analysis. In cases where multiple measures of the same cognitive construct were provided (e.g., ≥ 2 retrieval measures in a single study), the effect sizes were averaged to give each construct the same weight in the analysis. To check for differences in effect sizes between verbal and visual memory measurements, effect sizes for both dimensions were calculated; these were found not to differ significantly. This made it possible to include both verbal and visual memory measurements in the same analysis.

One study, Clague, Dudas, Thompson, Graham, and Hodges (2005), reported two different experiments. As it was unclear whether the same bvFTD sample was used in both experiments, only data from the first experiment were included in the meta-analysis. Ricci, Graef, Blundo, and Miller (2012) included an Italian and Australian bvFTD patient sample; these were included as two separate studies. Wicklund, Johnson, Rademaker, Weitner, and Weintraub (2006) and Lemos, Duro, Simoes, and Santana (2014) reported standard errors instead of standard deviations. We calculated the standard deviations based on the known confidence intervals and degrees of freedom.

The meta-analysis was performed in four consecutive steps. First, the overall effect size for patients with bvFTD *versus* control participants was calculated. Second, overall effect sizes for the four identified types of memory processes were calculated and compared between patients with bvFTD and controls. Third, the overall effect size for patients with bvFTD *versus* AD was calculated. Lastly, overall effect sizes for the four memory processes were calculated and compared between patients with bvFTD and AD.

Six pairwise comparisons were conducted between the four different types of memory processes. To check for the effect of differences in demographic features and dementia criteria between groups of studies on memory performance, additional analyses were performed with the demographic variables (age, education, gender, Mini Mental State Examination [MMSE]), type of bvFTD dementia criteria (Neary et al., 1998 or Rascovsky et al., 2011), and type of diagnosis (possible, probable, definite, mixed, or unknown) as categorical moderators. Rascovsky et al. (2011) revised the publication of consensus criteria by Neary et al. (1998) due to limitations. Among these were the ambiguity of behavioral descriptors, the inflexibility in applying the criteria (i.e., all five core features were required to manifest), and the insensitivity of the criteria in the early stages of the disease. The new criteria provide significant greater sensitivity (86%) than the 1998 criteria (53%). Age, education, percentage females, and MMSE were categorized as being either high or low, based on the median.

## RESULTS

**Fig. 1:**
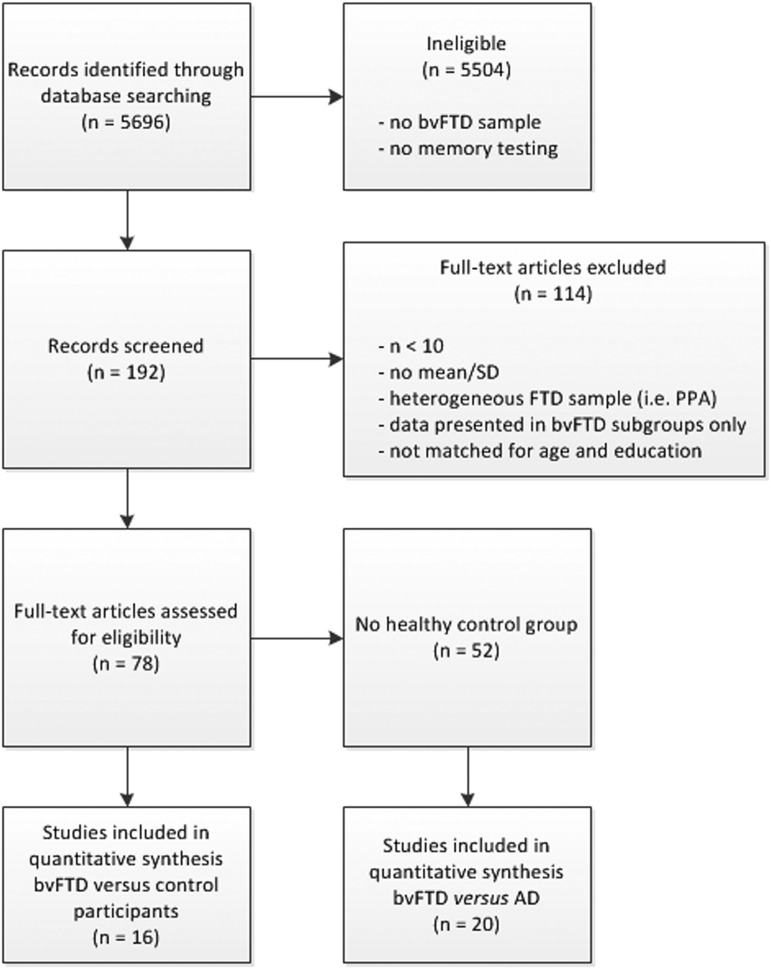
Flow chart illustrating the process of inclusion of eligible studies and reasons for exclusion.

In total, 16 studies comparing patients with bvFTD to healthy control participants and 20 studies comparing patients with bvFTD to patients with AD were included in the meta-analysis (Figure 1). Of these, 10 were included in both analyses as they included both a healthy control group and patients with AD. Tables 1 and 2 display the characteristics of these studies.

### Memory Performance in Patients With bvFTD *versus* Healthy Control Participants

#### Overall memory performance in bvFTD versus healthy controls

**Fig. 2:**
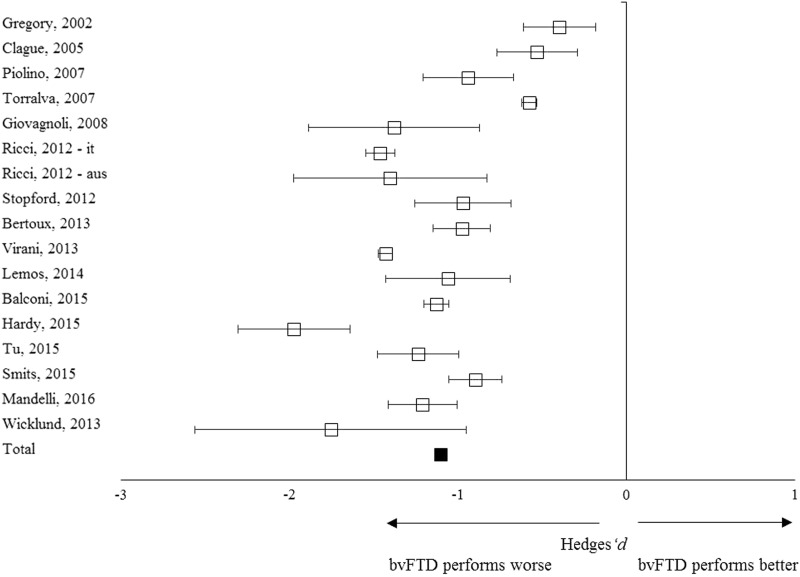
Forest plot illustrating effect sizes and bias-corrected 95% confidence intervals for each study comparing bvFTD patients to control participants on overall memory performance. Negative values indicate worse performance for bvFTD patients than for controls.

In total, 383 patients with bvFTD and 603 controls from 16 studies were included in the meta-analysis (Table 1). The overall weighed effect size for patients *versus* controls was –1.10 (95% CI [–1.23, –0.95]); % overlap=41.1 (Figure 2), indicating that patients performed significantly worse on overall memory performance than the controls. The test for heterogeneity was not significant (*Q*_*T*_=47.22; *p*=.34), suggesting that the variance among effect sizes was not greater than that expected by sampling error. The fail-safe number of studies was 4209.3, indicating that at least 4209 unpublished null-findings were needed to render the effects on memory statistically non-significant. It is unlikely that this number of unpublished studies with null effects relative to the published studies exists.

#### Working memory, learning, recall, and recognition memory in patients with bvFTD versus healthy controls

Working memory was assessed in eight studies and had an overall effect size of –0.83 (95% CI [–0.99, –0.63]); % overlap=48.4–52.6. Episodic memory learning was assessed in 14 studies with an overall effect size of –1.22 (95% CI [–1.50, –0.91]); % overlap 34.7–37.8. Episodic memory recall was assessed in 16 studies and showed an overall effect size of –1.15 (95% CI [–1.32, –0.95]); % overlap=37.8–41.1. Recognition memory was assessed in seven studies showing an overall effect size of –1.08 (95% CI [–1.49, –0.77]); % overlap=41.1–44.6. These effect sizes indicate worse performance on all memory processes in patients with bvFTD compared to controls. Despite a trend toward larger effect sizes for episodic memory learning and recall compared to working and recognition memory, the effect sizes were homogeneous, thereby indicating no statistically significant difference between the effect sizes of the four types of memory processes (*Q*_*M*_=4.32; *p*=.23).

### Memory Performance in Patients With bvFTD *versus* AD

#### Overall memory performance in bvFTD versus AD

**Fig. 3:**
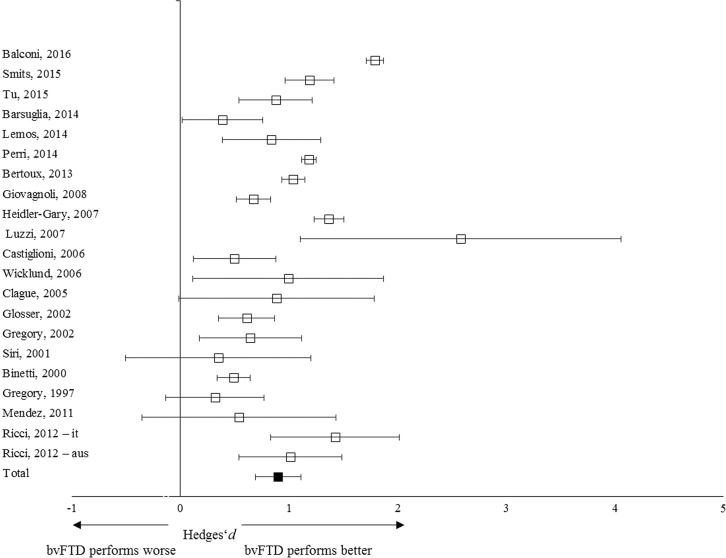
Forest plot illustrating effect sizes and bias-corrected 95% confidence intervals for each study comparing bvFTD patients to AD patients on overall memory performance. Positive values indicate better performance for the bvFTD patients than the AD patients.

A total of 452 patients with bvFTD and 874 with AD were included in the meta-analysis (Table 2). The overall weighed effect size for bvFTD *versus* AD was 0.85 (95% CI [0.69, 1.03]); % overlap=48.4–52.6. Patients with AD performed significantly worse than patients with bvFTD on overall memory performance (Figure 3). The heterogeneity test was significant (*Q*_*T*_=96.78; *p*<.01), indicating a possible moderating structure to the model (e.g., separate memory processes). The fail-safe number of studies was 3133.2, indicating that at least 3133 unpublished null-findings were needed to render the effects on memory statistically non-significant. It is unlikely that this number of unpublished studies with null effects relative to the published studies exists.

#### Working memory, learning, recall, and recognition memory in patients with bvFTD versus AD

Working memory was assessed in 11 studies with an overall effect size of 0.06 (95% CI [–0.12, –0.24]; % overlap>92.3). Episodic memory learning was assessed in 15 studies with an overall effect size of 1.00 (95% CI [0.78, 1.26]); % overlap=44.6. Episodic memory recall was assessed in 20 studies showing an overall effect size of 1.22 (95% CI [1.02, 1.51]); % overlap=37.8. Recognition memory was assessed in 5 studies with an overall effect size of 0.66 (95% CI [0.43, 0.87]); % overlap=57–61.8. These effect sizes indicate worse performance on learning and recall tests in patients with AD compared to those with bvFTD. AD patients had a slightly worse performance for recognition memory, but no differences in working memory was seen between patient groups.

This is corroborated by the heterogeneous Q-statistic results, indicating statistically significant differences between the effect sizes of the four memory processes (*Q*_*M*_=43.87; *p*<.01). Six pairwise comparisons showed significant differences between episodic memory recall and recognition memory (*Q*_*M*_=4.87; *p*=.027), between episodic memory recall and working memory (*Q*_*M*_=40.86; *p*<.01), between episodic memory learning and working memory (*Q*_*M*_=27.50; *p*<.01), and between working memory and recognition memory (*Q*_*M*_=7.93; *p*<.01).

### Moderator Variables

#### Patients with bvFTD versus control participants

The heterogeneity test for the bvFTD *versus* control studies showed no differences in effect sizes between older *versus* younger patients (*Q*_*M*_=1.11; *p*=.29), high-educated *versus* low-educated (*Q*_*M*_=0.81; *p*=.37), high *versus* low percentage of females (*Q*_*M*_=0.03; *p*=.85), and high *versus* low overall MMSE scores (*Q*_*M*_=3.58; *p*=.058). In addition, no significant differences were found in effect sizes between studies using different dementia criteria (Neary et al., 1998 or Rascovsky et al., 2011) (*Q*_*M*_=1.59; *p*=.21), or type of diagnosis (probable, definite, mixed, or unknown) (*Q*_*M*_=2.95; *p*=.39).

#### Patients with bvFTD versus AD

The heterogeneity test showed no differences in effect sizes between bvFTD *versus* AD studies with older *versus* younger (*Q*_*M*_=0.10; *p*=.75), high-educated *versus* low-educated (*Q*_*M*_ *=*1.19, *p*=.28), high *versus* low percentage of females (*Q*_*M*_=0.00; *p*=.99), high *versus* low MMSE score (*Q*_*M*_=0.07; *p*=.79). Furthermore, no differences were found based on type of dementia criteria used (Rascovsky et al., 2011, Neary et al., 1998 or DSM-IV/ICD-10) (*Q*_*M*_=1.46; *p*=.48), or type of diagnosis (possible, probable, definite, mixed or unknown) (*Q*= 3.83, *p*=.43).

## DISCUSSION

In this study, we conducted a meta-analytic review of memory in patients with bvFTD, to explore the extent, nature, and exact pattern of performance in these patients. The results showed large differences in memory performance between patients with bvFTD and controls and between patients with bvFTD and AD. This shows that patients with bvFTD perform at an intermediate level between healthy control participants and patients with AD. Nonetheless, patients with bvFTD show severe memory impairments across studies. Secondary analyses reveal significant differences in the four types of memory processes (i.e., working memory, episodic memory learning and recall, and recognition memory) when comparing bvFTD to AD. Learning and recall tests were found to be most discriminative, with recognition and working memory showing smaller to no discriminative power. This suggests that the patient groups can best be differentiated using learning and recall trials.

Our results are in line with previous studies reporting impaired memory in patients with bvFTD (e.g., Pennington, Hodges, & Hornberger, 2011; Simons et al., 2002), and those showing that patients with AD experience even greater memory problems (e.g., Frisoni et al., 1999; Galton et al., 2001; Hodges et al., 1999; Kramer et al., 2003; Kertesz, Davidson, McCabe, & Munoz, 2003; Lee, Rahman, Hodges, Sahakian, & Graham, 2003; Mathuranath, Nestor, Berrios, Rakowicz, & Hodges, 2000; Pachana et al., 1996; Pasquier et al., 2001; Perry & Hodges, 2000; Souliez, Pasquier, Lebert, Leconte, & Petite, 1996) with delayed memory testing being the most discriminative (e.g., Hutchinson & Mathias, 2007; Pasquier et al., 2001).

However, our results contrast with those of other studies reporting similar memory impairment in patients with bvFTD and AD (e.g., Hornberger et al., 2010; Pennington et al., 2011; Ranjith, Mathuranath, Sharma, & Alexander, 2010). Some of these authors argue for similar consolidation problems in patients with bvFTD and AD as damage to the hippocampal structures was visible in both groups (e.g., Barnes et al., 2006). Others theorize a selective retrieval disorder in patients with bvFTD, potentially caused by attention and executive problems (Glosser et al., 2002). They state that, because of disrupted attentional and executive control processes, patients with bvFTD may have difficulties generating strategies to encode and retrieve data from memory in an organized way (Glosser et al., 2002; Zakzanis, 1998).

The idea is that patients with bvFTD and AD do not differ in free recall measures, but that those with bvFTD would benefit from cued or recognition memory formats (Glosser et al., 2002). However, our results show a large difference in overall memory performance between patients with bvFTD and AD, with learning and recall tests being the most discriminative. Surprisingly, recognition memory yielded a smaller difference between the patient groups, suggesting that patients with bvFTD do not specifically benefit more from cued memory formats than those with AD. A possible explanation may be the limited number of studies including a recognition memory measure (*n*=5), but it may also be due to unsatisfactory psychometric characteristics of some of the measures such as RAVLT recognition memory (Schmidt, 1996).

Importantly, we report an overlap between 37% and 62% in the scores of the AD and bvFTD groups on episodic memory. This suggests that, even when the most discriminating memory measurements are used, the differential diagnosis of AD and bvFTD, on the basis of memory performance, remains challenging. These findings have clinical significance, as they suggest that performance on memory tests does not always adequately differentiate bvFTD from AD, thus questioning the inclusion of relative sparing as a diagnostic criterion for bvFTD diagnosis.

A possible explanation for the contrasting results in the literature and what we report here, supporting neither equal memory impairment in bvFTD and AD nor a sparing of episodic memory (as the current clinical criteria for bvFTD diagnosis suggest), could be the heterogeneity of bvFTD samples within and between studies. In approximately 30% of patients, FTD is caused by genetic mutations (e.g., progranulin [*GRN*], microtubule-associated protein tau [*MAPT*], and the chromosome 9 open reading frame 72 [*c9orf72*] repeat expansion). Ber et al. (2008) found a high frequency of episodic memory disorders (89%) in *GRN* mutation carriers and suggest an episodic memory disorder to be a distinctive characteristic of the *GRN* mutation, due to the high expression of *GRN* in the hippocampus in which marked atrophy and neuronal loss may be observed (Daniel, He, Carmichael, Halper, & Bateman, 2000; Boeve et al., 2006; Snowden et al., 2006).

However, Mahoney et al. (2012) have found similar results for *c9orf72* repeat expansion carriers, and suggest a similar explanation. It is, therefore, possible that the clinical presentation of memory impairment depends on the mutation involved. For example, Jiskoot et al. (2016) found specific recall deficits in presymptomatic *GRN* mutation carriers, whereas *MAPT* mutation carriers showed more prominent recognition deficits. Current and future longitudinal studies including neuropsychological testing should focus on investigating patterns of memory performance in different FTD phenotypes and their underlying pathologies. The development of tests that can disentangle the contributions of underlying pathology to memory impairment in bvFTD is highly recommended. Importantly, other memory processes such as autobiographical memory and future thinking have received increasing attention in recent years and seem to be valuable constructs to further address in future FTD research (e.g., Dermody, Hornberger, Piguet, Hodges, & Irish, 2016; Irish et al., 2018).

Strengths of our study include the use of a meta-analytical approach that provides a weighted estimate of the magnitude of effects. A limitation is the potential heterogeneity of the included studies with regards to the sample size and characteristics of the memory measurements. In addition, some of the secondary analyses included a relatively small number of studies. Importantly, the majority of the studies in this meta-analysis included patients with bvFTD without pathological confirmation. This introduces a potential selection bias based on the clinical criteria for bvFTD and AD. As relative sparing of episodic memory is considered an inclusion criterion for a bvFTD diagnosis, patients with memory impairment may have been misdiagnosed as AD or other forms of dementia, and were, therefore, not included in these studies.

Several recent clinicopathological studies have highlighted the risk of a misdiagnosis between AD and bvFTD (e.g., Graham et al., 2005; Womack et al., 2011). Although the Lund and Manchester criteria plus SPECT imaging results are considered to be acceptably accurate in identifying a clinical syndrome predicting the pathologic features of FTD at autopsy (Englund et al., 1994; Neary & Snowden, 1996), there is still the possibility that some of the studies missed patients with bvFTD with memory impairment due to the current clinical criteria. This selection bias would have led to an underestimation of our effect sizes.

We would like to stress, however, that several studies included pathologically proven patients with bvFTD and still found significant memory deficits (e.g., Bertoux et al., 2014; Graham et al., 2005; Hornberger et al., 2011). Moreover, by way of moderator analysis, we checked whether studies including pathologically proven patients with bvFTD differed in effect sizes on memory disorder from those that included possible or probable diagnoses or others where this was not specifically stated. Only a few studies included a definite bvFTD diagnoses (*n*=2), however, there was no significant difference in effect sizes.

In summary, our findings suggest that patients with bvFTD show large deficits on both working and episodic memory processes, with patients with AD performing worse on episodic memory. However, the overlap in test scores between the patient groups was too large to be able to make a confident differential diagnosis on the basis of memory performance. Therefore, we advise that clinicians use memory performances carefully, and interpret them in conjunction with other diagnostic information, that is, medical history, behavioral observations and questionnaires, neuroimaging, neuropsychological data of other cognitive domains. To improve on existing memory performance measures, we recommend developing tests that can disentangle the contribution of underlying pathology to memory impairment in bvFTD. Importantly, we show that memory impairment in bvFTD is more common than previously thought, thus it should not per definition be considered an exclusion criterion when diagnosing bvFTD.
